# Prevalence of sexual violence among refugees: a systematic review

**DOI:** 10.11606/s1518-8787.2019053001081

**Published:** 2019-09-12

**Authors:** Juliana de Oliveira Araujo, Fernanda Mattos de Souza, Raquel Proença, Mayara Lisboa Bastos, Anete Trajman, Eduardo Faerstein

**Affiliations:** I Universidade do Estado do Rio de Janeiro Instituto de Medicina Social Programa de Pós-Graduação em Saúde Coletiva Universidade do Estado do Rio de Janeiro. Instituto de Medicina Social. Programa de Pós-Graduação em Saúde Coletiva. Rio de Janeiro, RJ, Brasil; II Universidade Federal do Rio de Janeiro. Faculdade de Medicina. Programa de pós-Graduação em Clínica médica. Rio de Janeiro, RJ, Brasil.; III McGill University. Montreal, QC, Canadá.; IV Universidade do Estado do Rio de Janeiro. Instituto de Medicina Social. Departamento de Epidemiologia. Rio de Janeiro, RJ, Brasil

**Keywords:** Refugees, Sex Offenses, Rape, Review, Prevalence

## Abstract

**OBJECTIVE:**

To synthesize data about the prevalence of sexual violence (SV) among refugees around the world.

**METHODS:**

A systematic review was conducted from the search in seven bibliographic databases. Studies on the prevalence of SV among refugees and asylum seekers of any country, sex or age, whether in English, French, Spanish and Portuguese, were eligible.

**RESULTS:**

Of the 2,906 titles found, 60 articles were selected. The reported prevalence of SV was largely variable (0% to 99.8%). Reports of SV were collected in all continents, with 42% of the articles mentioning it in refugees from Africa (prevalence from 1.3% to 100%). The rape was the most reported SV in 65% of the studies (prevalence from 0% to 90.9%). The main victims were women in 89% of the studies, all the way, especially when still in the countries of origin. The SV was perpetrated particularly by intimate partners, but also by agents of supposed protection. Few studies have reported SV in men and children; the prevalence reached up to 39.3% and 90.9%, respectively. Approximately one-third of the studies (32%) were carried out in refugee camps and more than half (52%) in health services using mental health assessment tools. No study has addressed the most recent migratory crisis. Meta-analysis was not performed due to the methodological heterogeneity of the studies.

**CONCLUSIONS:**

SV is a prevalent problem affecting refugees of both sexes, of all ages, throughout the migratory journey, particularly those from Africa. Protection measures are urgently needed, and further studies, with more appropriate tools, may better measure the current magnitude of the problem.

## INTRODUCTION

The world is currently experiencing the biggest migratory crisis since World War II, with an increasing number of refugees. According to the United Nations High Commissioner for Refugees (UNHCR) report, 65.6 million people were forced to move because of persecution, conflict, widespread violence or human rights violations in 2016. Of these, 22.5 million were refugees; 2.8 million, asylum seekers; and 40.3 million, internally displaced persons within their own countries^1^.

Sexual violence (SV), defined as a sexual act or attempt to obtain a sexual act without the voluntary consent of the victim or with someone unable to consent or refuse^2^, is considered a present threat during forced displacement and the search for asylum^3,4^. In times of war, women and girls are more vulnerable to rape and are at greater risk for other forms of SV, such as early or forced marriage, intimate partner abuse, child sexual abuse, sexual exploitation and trafficking^4^. SV has also been perpetrated against men and boys as a tactic of war or during detention and interrogation^5^; they may suffer rape, sexual torture, mutilation, humiliation, enslavement, and forced incest^6^. This risk persists during the escape journey and after the reception in apparently safe destinations^7^.

The consequences can be extremely serious. In women, it can lead to mental disorders, obstetric complications, sexual dysfunctions, unwanted pregnancies, unsafe abortions and sexually transmitted infections^8,9^. Among men, in addition to infections and mental disorders, sexual dysfunction, somatic complaints, sleep disorders, withdrawal from relationships, attempted suicide, alcohol and drug abuse, and violent behavior are common^8,10^. In childhood, sexual abuse may also be accompanied by guilt, shame, eating disorders, cognitive distortions, mental disorders, sexual and relationship problems, and school absenteeism^11^.

Two previous systematic reviews have portrayed SV in refugees and internally displaced persons in emergency humanitarian complexes^12,13^: a meta-analysis aimed at estimating its prevalence in women only^12^, and other aimed at quantifying gender-based violence in three categories: physical violence, by intimate and sexual partner^13^. Neither analyzed the different types, profile of perpetrators and the moment of occurrence of SV in the migratory process. No studies have been conducted on the prevalence of this violence in the total refugee population (children, adults and older adults of both sexes) in different scenarios and moments of their trajectory, for a more comprehensive understanding of the magnitude of the problem.

Thus, we aim to synthesize the literature on the prevalence of SV in refugees around the world through a systematic review, regardless of sex, age and location. With this knowledge, one may better identify the profile of refugees who are victims of SV, contributing to specific prevention, approach, treatment and monitoring strategies in the countries of origin, during migration and in the host countries.

## METHODS

The bibliographic search was carried out in January 2018, using the MEDLINE (via Ovid), Embase (via Ovid), PsycINFO (via Ovid), Scopus, Web of Science, Sociological Abstracts (via ProQuest) and LILACS (via VHL) databases. No date limits or language restrictions were applied. Search strategies have involved the following MeSH and free terms: “refugee,” “asylum seek,” “exiled,” “refugee camps,” “sexual violence,” “sexual harassment,” “child abuse,” “sexual offense,” “sexual abuse,” “sexual crime,” “rape,” “sexual coercion,” “sexual assault.” Articles addressing any form of SV were included, using the connector “OR.” For the calculation by type of SV, we use the definition described in each of the articles. The search strategy is detailed in Appendix A. Articles within the bibliographic reference lists of the review studies and those included in this study were added where applicable.

Studies with data available for calculating the prevalence of SV in refugees or asylum seekers (considered as single population) in any country, sex or age, and published in English, French, Spanish and Portuguese were eligible. Chapters of books, dissertations, annals of congresses, editorials, letters, notes and comments were not included.

The selection of studies was initially conducted through the search of titles and abstracts; then by reading the full texts. Decisions on study eligibility and data extraction were performed by two independent reviewers on electronic forms constructed in EpiData 3.1 (EpiData Association, Odense, Denmark), and the differences were resolved by consensus or by a third reviewer. References were managed in EndNote Web software [Thomson Reuters (SCIENTIFIC), NY, USA].

Information was collected on: (1) study methods and population; (2) prevalence of SV according to sex, age, type of SV, continent/region/country of origin, host country/region, period of occurrence and profile of perpetrators.

In studies that presented additional categories of migrants (e.g. economic migrants), only information on refugees and asylum seekers was used. Likewise, in studies that reported psychological, physical and sexual violence, only SV data were used.

The calculation of global prevalence was estimated from the information on the total cases of the studies. For the calculation of specific prevalence, the following types of SV reported by the articles were considered: rape, attempted rape, unwanted sexual contact, non-contact unwanted sexual experience, sexual harassment, sexual abuse, sexual torture, sexual assault, sexual exploitation, including enforced prostitution and sex for survival, genital mutilation, forced marriage and abortion. When only the prevalence by type were informed and more than one of these forms was inflicted on the same victims, it was not possible to estimate the overall prevalence.

## RESULTS

**Figure 1:**
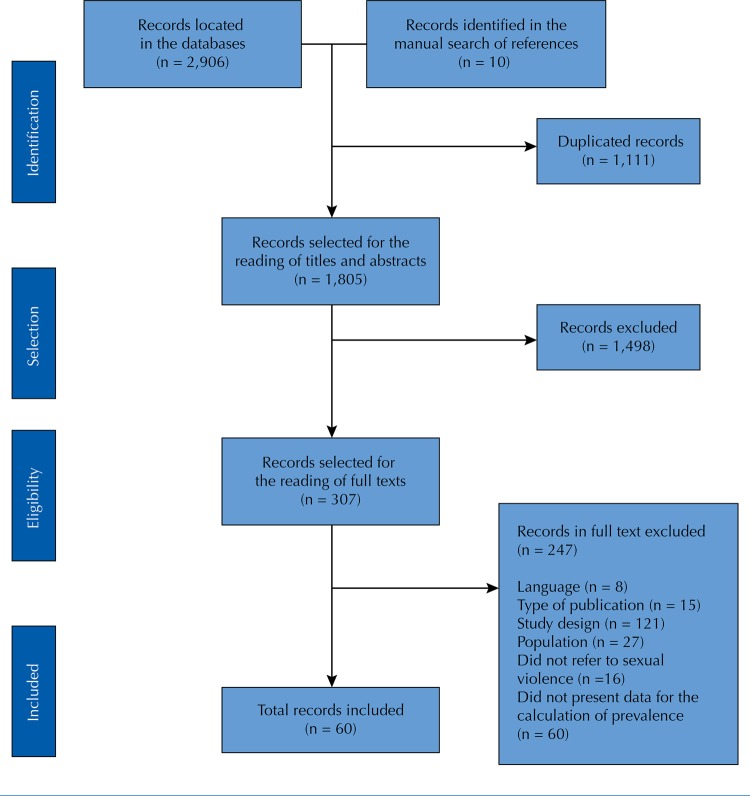
Flowchart for the selection of studies included in the systematic review.

We found 2,906 studies in the databases searched and 10 in the lists of bibliographic references (Figure 1). After the duplicates were removed (n = 1,111), 1,805 studies were selected for the reading of titles and abstracts. Of these, 1,498 were excluded by the following criteria: language (n = 29), type of publication (comments, letters, books, notes, editorials, abstracts of lectures and dissertations, n = 361), study design (most qualitative or review studies, n = 521), population not composed of refugees or asylum seekers (n = 176), out of scope (did not address SV, n = 131) or both (population and scope, n = 280).

Three hundred and seven studies were selected for the reading of full texts. After the application of the eligibility criteria, 60 studies were included for data extraction. Of the excluded ones, 15 were not original articles, 121 were review studies or with qualitative design and in 27 studies the population was not formed by refugees or asylum seekers.

### Characteristics of the Studies and their Populations

The 60 articles selected were all published in English between 1990 and 2017 (45% between 2000 and 2010) and from 31 different countries (14 from the USA). Studies were of cross-sectional design (Table 1), except for two cohort studies^48,73^.

**Table 1:** Characteristics of the studies included in the systematic review and prevalence of sexual violence. (n = 60)

First author and year of study	Country(ies)/ host region	Data collection location	Period of data collection		Instrument of study	Sampling (n)	Mean age of sample (years)	Female proportion (%)	Global SV prevalence (%)*	Prevalence of SV by sex	
	
			Start	End						Female	Male
Allodi^14^ (1990)	Canada	USS	1979	1985	NI	56	NI	50.0	NI	64.3	39.3
Fornazzari^15^ (1990)	Canada	USS	NI	NI	Collection in records	36	37	100.0	22.2	22.2	NA
Mckelvey^16^ (1995)	Philippines	USS	NI	NI	RQ	102	NI	33.3	9.8	8.8	10.3
Peel^17^ (1996)	United Kingdom	USS and detention centers	1993	1994	Collection in records	92	NI	21.7	33.7	80	20.8
Frljak^18^ (1997)	Bosnia and Herzegovina	USS	1993	1994	Collection in records	241	NI	100.0	3.3	3.3	NA
Silove^19^ (1998)	Australia	NI	NI	NI	HTQ	96	NI	NI	0.0	0.0	0.0
Gorst-Unsworth^20^ (1998)	United Kingdom	USS	NI	NI	HTQ	84	39	0.0	14.3	NA	14.3
Loutan^21^ (1999)	Switzerland	USS	1993	1994	HTQ	573	27	36.3	2.3	NI	NI
Blair^22^ (2000)	USA	USS and households	1991	1991	WTS	124	37	60.5	5.6	NI	NI
Hondius^23^ (2000)	Netherlands	USS	NI	NI	NI	156	NI	34	23.1	26.4	21.4
Petersen^24^ (2000)	Thailand	RC	1999	1999	RQ	129	36	37.2	NI	6.3	NI
Iacopino^25^ (2001)	Macedonia and Albania	RC	1999	1999	RQ	11,458	NI	NI	0.03	NI	NI
Tang^26^ (2001)	Gambia	RC	1999	1999	HTQ	80	41.3	48.8	1.3	NI	NI
Crescenzi^27^ (2002)	India	Villages	1995	1995	HTQ	150	NI	37.3	NI	NI	NI
Sabin^28^ (2003)	Mexico	RC	2000	2000	HTQ	170	37.9	58.2	3.5	NI	NI
Cardozo^29^ (2004)	Thailand	RC	2001	2001	HTQ	495	NI	57.4	NI	2.8	2.9
Sesay^30^ (2004)	Sierra Leone	RC and villages	2001	2011	RQ	400	NI	100.0	11.3	11.3	NA
Thomas^31^ (2004)	United Kingdom	NI	NI	NI	NI	100	16	41	32	63.4	10.2
Asgary^32^ (2006)	USA	USS	1998	2002	Istanbul Protocol	89	34	13.5	NI	NI	NI
Avdibegovic^33^ (2006)	Bosnia and Herzegovina	USS and RC	2000	2002	Modified DVI	50	NI	100.0	30.0	30.0	NA
Bradley^34^ (2006)	United Kingdom	USS	NI	NI	NI	97	30	14.4	8.2	28.6	2.4
Schweitzer^35^ (2006)	Australia	Community	2003	2003	HTQ	63	34.2	33.3	11.1	19	7.1
Olsen^36^ (2006)	Denmark	USS	1991	1994	RQ	221	35.6	12.7	11.3	NI	NI
Bogner^37^ (2007)	England	USS	2004	2005	RQ	27	NI	59.3	55.6	68.8	36.4
Edston^38^ (2007)	Sweden	USS	1993	2005	NI	63	28	100.0	76.2	76.2	NA
Hammoury^39^ (2007)	Lebanon	USS	2005	2005	AAS	349	28	100.0	26.4	26.4	NA
Hooberman^40^ (2007)	USA	USS	2000	2003	HTQ	325	33.5	38.8	28.9	NI	NI
John-Langba^41^ (2007)	Botswana	RC	NI	NI	SGBV	402	29.2	100.0	99.8	99.8	NA
Kira^42^ (2007)	USA	NI	NI	NI	CTS	501	35.7	45.3	1.2	NI	NI
Piwowarczyk^43^ (2007)	USA	USS	1999	2002	NI	134	34	65.7	50.0	NI	NI
Chang^44^ (2008)	USA	USS	2001	2001	NI	243	10.6	51.9	4.9	NI	NI
Nagai^45^ (2008)	Uganda	RC and villages	1999	2000	RQ	1,216	NI	78.0	NI	18.1	16.9
Harrison^46^ (2009)	Uganda	RC and villages	2006	2006	BSS	1,158	NI	52.4	NI	3.8	NI
Mitike^47^ (2009)	Ethiopia	RC	2004	2004	RQ	288	NI	100.0	42.4	42.4	NA
Williams^48^ (2010)	United Kingdom	USS	2005	2005	NI	178	30.4	35.4	25.8	54.0	10.4
Schubert^49^ (2011)	Finland	USS	NI	NI	HTQ	78	37.6	37.2	NI	NI	NI
Tamblym^50^ (2011)	USA	USS	2004	2007	HTQ modified	58	34.7	29.3	20.7	NI	NI
Bogic^51^ (2012)	Germany, Italy and United Kingdom	Households and communities	2005	2006	LSC	854	41.6	51.3	5.2	NI	NI
Kira^52^ (2012)	USA	NI	2006	2006	CTS	209	NI	0.0	90.9	NI	NI
Parmar^53^ (2012)	Republic of Cameroon	Villages	2010	2010	NI	191	35.1	100.0	40.8	40.8	NA
Black^54^ (2013)	USA	USS and Community	2004	2004	CREV SECV	196	13.8	45.9	4.6	NI	NI
Falb^55^ (2013)	Thailand	RC	2008	2008	RHA	861	30.1	100.0	NI	NI	NA
Tufan^56^ (2013)	Turkey	USS	2005	2007	SLESQ	67	30.6	41.8	20.9	46.4	2,6
Gibson-Helm^57^ (2014)	Australia	USS	2002	2011	NI	1,279	NI	100.0	5.7	5.7	NA
Idemudia^58^ (2014)	Polokwane, South Africa	City	NI	NI	RQ	125	28.3	42.3	NI	NI	NI
Morof^59^ (2014)	Uganda	NI	2010	2010	HTQ RQ	117	31.6	100.0	71.8	71.8	NA
Bell^60^ (2015)	Ruanda	RC	2008	2008	RHA toolkit	810	29	100.0	8.0	8.0	NA
Connor^61^ (2015)	USA	Community	NI	NI	RQ	30	31.8	100.0	93.3	93.3	NA
Sipsma^62^ (2015)	Ruanda	RC	NI	NI	RHA toolkit	548	32	100.0	38.1	38.1	NA
Al-Modallal^63^ (2016)	Jordan	RC	NI	NI	AAS	238	32.7	100.0	21.0	21.0	NA
Chu^64^ (2016)	USA	Communities and households	2014	2014	RQ	15	NI	100.0	60.0	60.0	NA
Lerner^65^ (2016)	USA	USS	2010	2013	RQ	267	34	33.0	33.3	NI	NI
Um^66^ (2016)	South Korea	NI	2010	2010	CTS2	180	39.8	100.0	25.6	25.6	NA
Wirtz^67^ (2016)	Ethiopia	RC	2012	2012	ASIST-GBV	487	NI	100.0	NI	NI	NA
Gušić^68^ (2017)	Sweden	Schools USS	NI	NI	WRGTI	77	NI	35.0	12.0	NI	NI
Hopkinson^69^ (2017)	USA	USS	2008	2013	HTQ RQ	61	28.8	37.7	62.3	NI	NI
Logie^70^ (2017)	Canada	Communities and social networks	2013	2015	RQ	42	NI	100.0	52.0	52.0	NA
Riley^71^ (2017)	Bangladesh	RC	NI	NI	HTQ	148	34	52.8	13.0	NI	NI
Stark^72^ (2017)	Ethiopia	RC	2015	2015	NI	919	14.6	100.0	65.3	65.3	NA
Wright^73^ (2017)	USA	Agencies of settlement	2011	2012	HTQ	298	NI	45.0	NI	1.5	NI

SV: sexual violence; NA: not applicable; NI: not informed; RC: refugee camps; USA: Unites States of America; USS: health services units; RQ: research questionnaire; HTQ: Harvard Trauma Questionnaire; STAR: Resettlement Stressor Scale; WTS: War Trauma Scale; DVI: Domestic Violence Inventory; AAS: Abuse Assessment Screen; SBGV: Sexual and Gender-based Violence Scale; CTS: Revised Conflict Tactics Scales; CREV: Children’s Report of Exposure to Violence; SECV: Survey of Exposure to Community Violence; BSS: Behavioral Surveillance Surveys Questionnaire; SLESQ: Stressful Life Events Screening Questionnaire; LEC: Life Events Checklist; ASIST-GBV: Assessment Screen to Identify Survivors Toolkit for Gender Based Violence; LSC: Life Stressor Checklist; RHA: Reproductive Health Assessment; WRGTI: War/refugee and general trauma inventory

* The global prevalence of SV was calculated from the total number of cases reported by the studies or, when there was no such information, by the total sum of the specific cases reported (e.g. cases of rape, sexual harassment, etc.). However, in five studies^32,49,55,58,67^, the global prevalence could not be estimated since the authors did not report the total number of cases. It was not possible to calculate it from the sum of the typified prevalence because there were victims who suffered more than one type of SV, which would overestimate the calculation of the global prevalence.

The most frequent sites of data collection, according to the 54 articles that contained this information, were health services (n = 28.52%) and refugee camps (n = 17.32%). Most studies (87%) were conducted to evaluate outcomes in mental health, without the main objective of measuring the prevalence of SV cases. Among the 49 studies that informed the instrument used, the Harvard Trauma Questionnaire (HTQ) was the most frequently validated instrument (n = 15, corresponding to 31%), while 29% (n = 14) used questionnaires designed specifically for the research.

Studies involved 28,101 refugees and asylum seekers. The population of each study varied between 15 and 11,458 individuals. In 33% (n = 20) of the studies, the sample included less than 100 people, and in 18% (n = 11), more than 500 people. The mean age of participants ranged from 10.6 to 41.6 years old; 42% (n = 25) of the studies included those younger than 18 years. There was a general predominance of women; in 37% (n = 21) of the studies, the sample was exclusively female. The predominant religion was Muslim, in 12 (63%) of the 19 studies with data about it.

### Prevalence of Sexual Violence

The global prevalence variation presented a large amplitude, regardless of the sample size: from 0% to 99.8%, with a total of 2,859 cases of SV. In 15 studies (31%), the prevalence was less than 10% (samples from 80 to 11,458 people), and in 11 (23%), more than 50% (samples from 15 to 919 people), as shown in Table 1. This wide variation occurred independently of the data collection scenario – in refugee camps (n = 12, 0.03% to 99.8%), health units (n = 25, 2.3% to 76.2%) and communities/villages (n = 6, 5.2% to 93.3%) – and assessing form – validated instruments (n = 25%; 0.0% to 99.8%) or questionnaires of the own research (n = 14; 0.03% to 93.3%).

Six studies reported SV in children and adolescents, with prevalence varying between 4.6% and 90.9%^16,44,47,52,54,72^. In 32 of the 36 (89%) studies that showed prevalence by sex, the main victims were women. Of these, 12 studies reported SV in both sexes, with a difference of up to 59.2% more of prevalence in women^17^. Two studies reported the opposite, but with disparities less than 2%^16,29^. In men, the prevalence reached 39.3%^14^.

Africa was the most frequent continent of origin in 13 (42%) of the 31 studies with information about it (Table 2). As to the moment of occurrence, approached by 18 studies, 17 (94%) reported that SV occurred in the country of origin (prevalence between 1% and 92%); in two studies (11%), it occurred during the course (prevalence of 5.2% in both)^53,68^; and two (11%) reported SV at the host site (prevalence of 39% in Cameroon^53^ and 46.1% in Uganda^59^).

**Table 2:** Prevalence of sexual violence in refugees according to place of origin. (n = 31)

Continent(s) of origin	Region of origin	Country of origin	First author and year of study	Sampling (n)	Prevalence of SV
Africa (n = 13)	NI	NI	Thomas^31^ (2004)	65	24.6
	NI	NI	Chu^64^ (2016)	15	60.0
	Central Africa	RDC	Peel^17^ (1996)	92	33.7
		RDC	Edston^38^ (2007)	3	100.0
		Central African Republic	Parmar^53^ (2012)	77	57.1
		RDC	Bell^60^ (2015)	810	8.0
		RDC	Sipsma^62^ (2015)	548	38.1
	West Africa	Senegal	Tang^26^ (2001)	80	1.3
		Sierra Leone	Sesay^30^ (2004)	400	11.3
		NI	Gibson-Helm^57^ (2014)	45	6.7
	North Africa	Sudan	Schweitzer^35^ (2006)	63	11.1
		Sudan and South Sudan	Stark^72^ (2017)	919	65.3
		NI	Gibson-Helm^57^ (2014)	1,147	5.1
	East Africa	Uganda	Edston^38^ (2007)	9	66.7
		Somalia	Mitike^47^ (2009)	248	49.2
		NI	Gibson-Helm^57^ (2014)	87	13.8
Asia (n = 8)	Southern Asia	Sri Lanka	Silove^19^ (1998)	92	0.0
		Bangladesh	Edston^28^ (2007)	13	84.6
	South Asia	Myanmar	Petersen^24^ (2000)	129	2.3
		Myanmar	Riley^71^ (2017)	148	13.0
	Southeastern Asia	Vietnam	McKelvey^16^ (1995)	102	9.8
		Cambogia	Blair^22^ (2000)	124	5.6
		Cambogia	Chang^44^ (2008)	243	4.9
	East Asia	North Korea	Um^66^ (2016)	180	25.6
Europe Asia Africa (n = 8)	Middle East	NI	Olsen^36^ (2006)	221	11.3
		NI	Wright^73^ (2017)	133	1.5
Europe Asia Africa (n = 8)	Middle East	Iraq	Gorst-Unsworth^20^ (1998)	84	14.3
		Iraq	Kira^42^ (2007)	501	1.2
		Iraq	Kira^52^ (2012)	209	90.9
		Iraq	Black^54^ (2013)	196	4.6
		Iran	Edston^38^ (2007)	11	45.5
		Syria	Edston^38^ (2007)	3	66.7
		Turkey	Bradley^34^ (2006)	97	8.2
		Turkey	Edston^38^ (2007)	3	100.0
NA (n = 2)	Palestine	NA	Hammoury^39^ (2007)	349	26.4
		NA	Al-Modallal^63^ (2016)	238	21.0
America (n = 1)	Central America	Guatemala	Sabin^28^ (2003)	170	3.5
Europe (n = 1)		Bosnia	Frljak^18^ (1997)	241	3.3

SV: sexual violence; NI: not informed; NA: not applicable; DRC: Democratic Republic of the Congo

The most frequent type of SV was rape (65%) (Table 3). The perpetrators were identified in 18 studies: 10 (55%) reported the occurrence of SV by intimate partner (prevalence from 4.3% to 30%)^33,39,45,53,55,59,62,63,66,72^, five by military personnel (prevalence from 1% to 74.6%)^38,45,55,58,72^, four by acquaintances^51,53,55,72^, four by relatives^45,54,58,72^, two by unknowns ^51,53^, two by rebel soldiers ^31,53^, one by police officers^58^, one by armed groups^72^, and one by guards in prison^17^.

**Table 3:** Prevalence according to the type of sexual violence in refugees. (n = 51)

Type of sexual violence	First author and year of study	Continent/region/country of origin	Host country/region	Prevalence (%)
Rape (n = 33)	Allodi^14^ (1990)	Latin America	Canada	30.4
	Fornazzari^15^ (1990)	Latin America	Canada	22.2
	Peel^17^ (1996)	RDC	United Kingdom	33.7
	Frljak^18^ (1997)	Bosnia	Bosnia	3.3
	Silove^19^ (1998)	Sri Lanka	Australia	0.0
	Loutan^21^ (1999)	Africa, Asia and Europe	Switzerland	2.3
	Petersen^24^ (2000)	Myanmar	Thailand	2.3
	Tang^26^ (2001)	Senegal	Gambia	1.3
	Crescenzi^27^ (2002)	Tibet	India	0.7
	Cardozo^29^ (2004)	Myanmar	Thailand	2.8
	Sesay^30^ (2004)	Sierra Leone	Sierra Leone	11.3
	Thomas^31^ (2004)	Africa, Middle East, Western Europe and Asia	United Kingdom	32.0
	Asgary^32^ (2006)	Africa and Asia	USA	6.7
	Bradley^34^ (2006)	Turkey	United Kingdom	1.0
	Schweitzer^35^ (2006)	Sudan	Australia	11.1
	Avdibegovic^33^ (2006)	NI	Bosnia	34.0
	Bogner^37^ (2007)	Middle East, Europe, Africa and Latin America	England	44.4
	Edston^38^ (2007)	Africa, Asia and Middle East	Sweden	76.2
	Hammoury^39^ (2007)	Palestine	Lebanon	26.4
	Hooberman^40^ (2007)	Africa, Asia, Europe and Central and South America	USA	18.2
	Harrison^46^ (2009)	Africa	Uganda	2.0
	Williams^48^ (2010)	Africa and Middle East	United Kingdom	16.3
Rape (n = 33)	Schubert^49^ (2011)	Middle East, Southeast Europe, South Asia and Central Africa	Finland	21.8
	Kira^52^ (2012)	Iraq	USA	90.9
	Falb^55^ (2013)	Myanmar	Thailand	0.3
	Morof^59^ (2014)	Somalia and DRC	Uganda	54.7
	Idemudia^58^ (2014)	Zimbabwe	Polokwane, South Africa	56.8
	Bell^60^ (2015)	RDC	Ruanda	8.0
	Lerner^65^ (2016)	Africa, America and Western Europe	USA	33.3
	Wirtz^67^ (2016)	Somalia	Ethiopia	20.1
	Hopkinson^69^ (2017)	Africa, Asia, America and Eastern Europe	USA	42.6
	Logie^70^ (2017)	NI	Canada	52.0
	Stark^72^ (2017)	Sudan and South Sudan	Ethiopia	16.1
Unwanted sexual contact (n = 7)	Asgary^32^ (2006)	Africa and Asia	USA	6.7
	Avdibegovic^33^ (2006)	NI	Bosnia	2.0
	Schubert^48^ (2011)	Middle East, Southeast Europe, South Asia and Central Africa	Finland	46.2
	Falb^55^ (2013)	Southeastern Asia	Thailand	0.7
	Idemudia^58^ (2014)	Zimbabwe	Polokwane, South Africa	63.2
	Hopkinson^69^ (2017)	Africa, Asia, America and Eastern Europe	USA	24.6
	Stark^72^ (2017)	Sudan and South Sudan	Ethiopia	22.0
Sexual coercion (n = 1)	Stark^72^ (2017)	Sudan and South Sudan	Ethiopia	27.3
Attempted rape (n = 2)	Idemudia^58^ (2014)	Zimbabwe	Polokwane, South Africa	44.8
	Morof^59^ (2014)	Somalia and DRC	Uganda	64.1
Forced pregnancy (n = 1)	Wirtz^67^ (2016)	East Africa	Ethiopia	15.6
Sexual torture (n = 6)	Hondius^23^ (2000)	Turkey and Iran	Netherlands	23.1
	Asgary^32^ (2006)	Africa and Asia	USA	9.0
	Bradley^34^ (2006)	Turkey	United Kingdom	2.1
	Olsen^36^ (2006)	Middle East	Denmark	11.3
	Bogner^37^ (2007)	Middle East, Europe, Africa and Latin America	England	11.1
	Tamblyn^50^ (2011)	Africa	USA	20.7
Sexual Assault (n = 5)	Gorst-Unsworth^20^ (1998)	Iraq	United Kingdom	14.3
	Iacopino^25^ (2001)	Kosovo	Macedonia	0.03
	Bradley^34^ (2006)	Turkey	United Kingdom	8.2
	Hooberman^40^ (2007)	Africa, Asia, Central and South America and Europe	USA	10.8
	Williams^48^ (2010)	Africa and Middle East	United Kingdom	12.9
Genital mutilation (n = 6)	Asgary^32^ (2006)	Africa and Asia	USA	2.2
	Bradley^34^ (2006)	Turkey	United Kingdom	1.0
	Mitike^47^ (2009)	Somalia	Ethiopia	42.4
	Gibson-Helm^57^ (2014)	Africa and Middle East	Australia	5.7
	Connor^61^ (2015)	Somalia and Ethiopia	USA	93.3
	Chu^64^ (2016)	Africa	USA	60.0
Sexual exploitation (n = 4)	Cardozo^29^ (2004)	Myanmar	Thailand	1.0
	Nagai^45^ (2008)	Sudan	Uganda	82.0
	Idemudia^58^ (2014)	Zimbabwe	South Africa	44.0
	Wirtz^67^ (2016)	Somalia	Ethiopia	27.3
Non-contact unwanted sexual experiences (n = 5)	Crescenzi^27^ (2002)	Tibet	India	24.6
	Asgary^32^ (2006)	Africa and Asia	USA	4.5
	Avdibegovic^33^ (2006)	NI	Bosnia	2.0
	Falb^55^ (2013)	Myanmar	Thailand	1.5
	Hopkinson^69^ (2017)	Africa, Asia, America and Eastern Europe	USA	29.8
Sexual Abuse (n = 8)	Allodi^14^ (1990)	Latin America	Canada	21.4
	McKelvey^16^ (1995)	Vietnam	Philippines	9.8
	Blair^22^ (2000)	Cambodia	USA	5.6
	Kira^42^ (2007)	Iraq	USA	1.2
	Chang^44^ (2008)	Cambodia	USA	4.9
	Nagai^45^ (2008)	Sudan	Uganda	85.0
	Black^54^ (2013)	Iraq	USA	4.6
	Riley^71^ (2017)	Myanmar	Bangladesh	13.0
Forced marriage (n = 2)	Asgary^32^ (2006)	Africa and Asia	USA	2.2
	Wirtz^67^ (2016)	Somalia	Ethiopia	19.5
Sexual Harassment (n = 4)	Asgary^32^ (2006)	Africa and Asia	USA	12.4
	Bogic^51^ (2012)	Bosnia	Germany, Italy and United Kingdom	5.2
	Idemudia^58^ (2014)	Zimbabwe	Polokwane, South Africa	52.8
	Wright^73^ (2017)	Middle East	USA	1.5

NI: not informed; USA: United States of America; DRC: Democratic Republic of the Congo

In five studies^32,49,55,58,67^, the authors did not report the number of victims, and it was not possible to estimate the overall prevalence. Estimating the sum of prevalence by specific type would overestimate the overall prevalence due to cases that suffered more than one type of SV.

## DISCUSSION

Previous studies have shown that SV is a constant threat throughout the refugee migration pathway^3,12,13^, which has been confirmed in the present review. Although most of the studies identified here revealed a higher prevalence among adult women, SV was also a serious problem in men and children. In addition, we observed the SV is perpetrated mainly by intimate partners, but also by military, guards and police. Most cases occur in the country of origin, in the form of rape and in refugees from Africa. In some refugee camps, such as Uganda and Cameroon, the frequency was alarming.

It is possible that prevalence may be underestimated in some studies, since many victims – especially men – do not report SV because of shame, threats by perpetrators, fear of being found guilty or suffering from stigma and exclusion from family and community^6,74^, with consequent low demand for health care and case records^75^. In addition, the humanitarian crisis caused by armed conflicts in the refugees’ countries of origin leads to large displacements of people and demands incompatible with the availability of health services and resources^76^, which may further reduce the chances of case identification. On the other hand, studies focused on the evaluation of mental trauma in health services may overestimate the prevalence.

In the meta-analysis of SV prevalence in women in emergency humanitarian complex scenarios, which also included internally displaced persons and excluded genital mutilation, the mean prevalence was 21.4% and higher in refugees from Africa^12^. In our review, we found several studies with a much higher prevalence. Regardless of the actual prevalence, SV was frequent in the populations studied, and deserves special attention in the health services and the reception of this population already weakened by traumas of war and persecution.

Young women are the main victims of SV, but men, children and adolescents are also victims, a reality little discussed in the literature. Men and unaccompanied minors are also exposed to the risk of sexual exploitation and abuse during migration and arrival in destination countries^3^. Nevertheless, the predominance in women is not surprising. The immigration process is accompanied by difficulties such as economic insecurity, language barriers and acculturation, which lead to the imbalance of power between women and partners, leading to increased tensions^77^. Because of economic, political, and social changes during wars and postwar periods, many men use violence to control women and reestablish their status of power^78^. Such conditions may explain the higher frequency of SV perpetrated by intimate partners.

SV occurs mainly before migration, in the countries of origin of the refugees. This suggests a relation with the conditions generated by the armed conflicts, which potentiate cultural norms of superiority of the masculine power present in these places, even before the condition of search of refuge. High prevalence in Africa supports this view. The Democratic Republic of Congo, where armed conflicts over natural resource reserves have lasted since independence in 1960^79^, is marked by atrocities including group rape, sexual slavery, forced family involvement in rape, genital mutilation, among others^80^. More shocking is the fact that, even when hosted in refugee camps, this already fragile population still faces insecurity and suffers SV perpetrated by those from whom they expect protection, such as officers and police.

Rape was the most mentioned form of this violence. This can be explained by the more concrete definition, by the most remarkable experience, and because most studies have used the HTQ instrument, which has a specific question about rape and sexual abuse, but not about other forms of SV. Rape is considered the cruelest type because it brings serious and severe consequences to the health of the victims. War survivors diagnosed with posttraumatic stress disorder and rape victims report more somatic symptoms than those without a rape experience^81^. Rape also increases the chances of acquiring HIV infection, as reported in sub-Saharan African refugee women in Paris, and is related to social difficulties and lack of fixed residence due to the risk of transactional sex or sexual harassment during lodging by relatives or acquaintances^82^.

Several studies included in this review had many limitations, such as lack of detail on the population, outcome of interest, timing of the occurrence, profile of the perpetrators, gender and age of the victims. In addition, the studies did not include victims of the most recent migratory crisis, which began in 2015.

Our review also has limitations. The literature search did not include the terms “sexual torture” and “genital mutilation,” which may have resulted in low sensitivity and explained the number of articles found in reference lists. We did not include the gray literature and no methodological quality evaluation of the selected studies was performed. In addition, we did not restrict the sample size of the articles, which resulted in imprecise estimates in studies with few individuals^38^. Finally, methodological differences between the studies (different data collection sites, such as mental health services and refugee camps; different data collection instruments; studies focusing on mental disorders rather than SV prevalence; and unequal sampling) have contributed to the diversity of the rates found and heterogeneity between the studies, which prevented a meta-analysis to summarize the information.

In summary, results of this review show that SV is a frequent problem among refugees, both women and men, mainly those from Africa, and occurs at all times in the migratory process, including in places of supposed reception and protection. The SV problem among refugees from the most recent migratory crisis must be investigated in unselected scenarios and with more appropriate methods to better guide the necessary protection measures.
