# Supplementary material for: Prevalence of sexual violence among refugees: a systematic review
Source: Rev Saude Publica. 2019 Sep 12;53:78. doi: 10.11606/s1518-8787.2019053001081 (PMC6752644; doi:10.11606/s1518-8787.2019053001081)
Supplement: Translation

# Translation: Prevalência de violência sexual em refugiados: uma revisão sistemática

**Authors:** Juliana de Oliveira Araujo, Fernanda Mattos de Souza, Raquel Proença, Mayara Lisboa Bastos, Anete Trajman, Eduardo Faerstein

**Affiliations:** I Universidade do Estado do Rio de Janeiro. Instituto de Medicina Social. Programa de Pós-Graduação em Saúde Coletiva. Rio de Janeiro, RJ, Brasil; II Universidade Federal do Rio de Janeiro. Faculdade de Medicina. Programa de pós-Graduação em Clínica médica. Rio de Janeiro, RJ, Brasil.; III McGill University. Montreal, QC, Canadá.; IV Universidade do Estado do Rio de Janeiro. Instituto de Medicina Social. Departamento de Epidemiologia. Rio de Janeiro, RJ, Brasil

**Keywords:** Refugiados, Delitos Sexuais, Estupro, Revisão, Prevalência

## Abstract

**OBJETIVO:**

Sintetizar dados da literatura sobre a prevalência de violência sexual em refugiados.

**MÉTODOS:**

Conduzimos uma revisão sistemática a partir da busca em sete bases bibliográficas. Foram elegíveis estudos em inglês, francês, espanhol e português com dados sobre a prevalência de violência sexual em refugiados e requerentes de asilo, de qualquer país, sexo ou idade.

**RESULTADOS:**

Dos 2.906 títulos encontrados, 60 artigos foram incluídos. A prevalência foi amplamente variável (0% a 99,8%). Houve relatos de violência sexual em todos os continentes, com 42% dos artigos mencionando-a em refugiados provenientes da África (prevalências de 1,3% a 100%). O estupro foi a ocorrência mais relatada em 65% dos estudos (prevalências de 0% a 90,9%). As principais vítimas foram mulheres em 89% dos estudos, em todo o trajeto, principalmente nos países de origem. A violência foi perpetrada particularmente por parceiros íntimos, mas também por agentes de suposta proteção. Poucos estudos relataram-na em homens e crianças, com prevalências atingindo até 39,3% e 90,9%, respectivamente. Cerca de 1/3 dos estudos (32%) foram realizados em campos de refugiados ou locais de acolhimento, e mais da metade (52%) em serviços de saúde, utilizando instrumentos de avaliação de saúde mental. Nenhum estudo abordou a crise migratória mais recente. Não foi realizada meta-análise devido à heterogeneidade metodológica dos estudos.

**CONCLUSÕES:**

A violência sexual é um problema prevalente que atinge refugiados de ambos os sexos, de todas as idades, em particular aqueles provenientes da África, durante todo o percurso migratório. Medidas de proteção são urgentemente necessárias, e novos estudos, com instrumentos mais apropriados, poderão mensurar melhor a magnitude atual do problema.

## INTRODUÇÃO

Atualmente, o mundo vivencia a maior crise migratória desde a Segunda Guerra Mundial, com o aumento crescente do número de refugiados. De acordo com o relatório do Alto Comissariado das Nações Unidas para Refugiados (ACNUR), 65,6 milhões de pessoas foram forçadas a se deslocar em razão de perseguição, conflito, violência generalizada ou violações de direitos humanos em 2016. Desse total de migrantes, 22,5 milhões eram refugiados; 2,8 milhões, solicitantes de refúgio; e 40,3 milhões, deslocados internos dentro de seus países^1^.

A violência sexual (VS), definida como ato sexual ou tentativa de obter um ato sexual sem o consentimento voluntário da vítima ou com alguém incapaz de consentir ou recusar^2^, é considerada uma ameaça presente durante o deslocamento forçado e a busca de asilo^3,4^. Em tempos de guerra, mulheres e meninas são mais vulneráveis a estupros e apresentam maior risco de sofrer outras formas de VS, como casamento precoce ou forçado, abuso por parceiro íntimo, abuso sexual infantil, exploração e tráfico sexual^4^. A VS também tem sido perpetrada contra homens e meninos como tática de guerra ou durante a detenção e interrogatórios^5^; eles podem sofrer estupro, tortura sexual, mutilação, humilhação, escravização e incesto forçado^6^. Esse risco persiste durante a jornada de fuga e após o acolhimento em destinos aparentemente seguros^7^.

As consequências podem ser extremamente graves. Nas mulheres, pode levar a distúrbios mentais, complicações obstétricas, disfunções sexuais, gestações indesejadas, abortos inseguros e infeções sexualmente transmissíveis^8,9^. Entre homens, além das infecções e transtornos mentais, são comuns a disfunção sexual, queixas somáticas, distúrbios do sono, afastamento de relacionamentos, tentativa de suicídio, abuso de álcool e drogas e comportamento violento^8,10^. Na infância, o abuso sexual pode ser acompanhado também de culpa, vergonha, distúrbios alimentares, distorções cognitivas, distúrbios mentais, problemas sexuais e de relacionamento e absenteísmo escolar^11^.

Duas revisões sistemáticas prévias retrataram a VS em refugiados e deslocados internos em complexos humanitários de emergência^12,13^: uma revisão com meta-análise buscou estimar a prevalência somente em mulheres^12^, e a outra objetivou quantificar a violência baseada no gênero em três categorias: violência física, por parceiro íntimo e sexual^13^. Nenhuma analisou os diferentes tipos, perfil dos perpetradores e momento da ocorrência da VS no processo migratório. Ainda não há estudos sobre a prevalência dessa violência na população total de refugiados (crianças, adultos e idosos de ambos os sexos) em diferentes cenários e momentos do percurso, para uma compreensão mais abrangente da magnitude do problema.

Assim, objetivamos sintetizar a literatura sobre a prevalência de VS em refugiados no mundo por meio de uma revisão sistemática, independentemente do gênero, idade e local onde se encontram. Com esse conhecimento, será possível identificar melhor o perfil dos refugiados vítimas de VS, contribuindo para estratégias específicas de prevenção, abordagem, tratamento e monitoramento nos países de origem, durante o percurso migratório e nos países de acolhimento.

## MÉTODOS

A busca bibliográfica foi realizada em janeiro de 2018, nas bases MEDLINE (via Ovid), Embase (via Ovid), PsycINFO (via Ovid), Scopus, Web of Science, Sociological Abstracts (via ProQuest) e LILACS (via BVS). Não foram aplicados limites de data ou restrição de idioma. As estratégias de buscas envolveram os seguintes termos MeSH e livres: “*refugee*”*,* “*asylum seek*”*,* “*exiled*”*,* “*refugee camps*”*,* “*sexual violence*”*,* “*sexual harassment*”*,* “*child abuse*”*,* “*sexual offense*”*,* “*sexual abuse*”*,* “*sexual crime*”*,* “*rape*”*,* “*sexual coercion*”*,* “*sexual assault*”. Artigos que abordassem qualquer forma de VS foram incluídos, com a utilização do conector “OU”. Para o cálculo por tipo de VS, utilizamos a definição descrita em cada um dos artigos. A estratégia de busca está detalhada no apêndice A. Artigos presentes nas listas de referências bibliográficas dos estudos de revisão e daqueles incluídos no presente estudo foram adicionados, quando aplicável.

Foram elegíveis estudos com dados disponíveis para o cálculo da prevalência de VS em refugiados ou requerentes de asilo (considerados como população única) em qualquer país, sexo ou idade e publicados em inglês, francês, espanhol e português. Não foram incluídos capítulos de livros, dissertações, anais de congressos, editoriais, cartas, notas e comentários.

A seleção dos estudos foi inicialmente conduzida pela consulta de títulos e resumos; em seguida, pela leitura dos textos completos. As decisões sobre elegibilidade dos estudos e extração dos dados foram realizadas por dois revisores independentes, em formulários eletrônicos construídos no EpiData 3.1 (EpiData Association, Odense, Dinamarca), e as divergências foram resolvidas por consenso ou pela avaliação de um terceiro revisor. As referências foram gerenciadas no *software* EndNote Web [Thomson Reuters (SCIENTIFIC), NY, EUA].

Foram coletadas informações sobre: (1) métodos e população do estudo; (2) prevalência de VS, de acordo com sexo, idade, tipo de VS, continente/região/país de origem, país/região de acolhimento, período da ocorrência e perfil dos perpetradores.

Nos estudos que apresentaram outras categorias adicionais de migrantes (por exemplo, migrantes econômicos), foram utilizadas somente as informações relativas a refugiados e requerentes de asilo. Da mesma forma, em estudos que relataram violência psicológica, física e sexual, foram utilizados somente os dados relativos à VS.

O cálculo da prevalência global foi estimado a partir das informações sobre o total de casos dos estudos. Para o cálculo das prevalências específicas, foram considerados os seguintes tipos de VS informados pelos artigos: estupro, tentativa de estupro, contato sexual indesejado, experiência sexual indesejada sem contato, assédio sexual, abuso sexual, tortura sexual, agressão sexual, exploração sexual incluindo prostituição forçada e sexo por sobrevivência, mutilação genital, casamento e aborto forçados. Quando apenas as prevalências por tipo estavam informadas e mais de uma dessas formas foi infligida às mesmas vítimas, não foi possível estimar a prevalência global.

## RESULTADOS

Foram encontrados 2.906 estudos nas bases de dados pesquisadas e 10 nas listas de referências bibliográficas (Figura 1). Após a remoção das duplicatas (n = 1.111), 1.805 estudos foram selecionados para leitura dos títulos e resumos. Desses, foram excluídos 1.498 pelos seguintes critérios: idioma (n = 29), tipo de publicação (comentários, cartas, livros, notas, editoriais, resumos de conferências e dissertações, n = 361), desenho de estudo (em grande parte estudos qualitativos e de revisão, n = 521), população não composta por refugiados ou requerentes de asilo (n = 176), fora do escopo (não abordavam VS, n = 131) ou ambos (população e escopo, n = 280).

**Figura 1:** Fluxograma de seleção dos estudos incluídos na revisão sistemática.

Trezentos e sete estudos foram selecionados para leitura dos textos completos. Após a aplicação dos critérios de elegibilidade, foram incluídos 60 estudos para a extração dos dados. Dentre os excluídos, 15 não eram artigos originais, 121 eram estudos de revisão ou com desenho qualitativo e em 27 estudos a população não era formada por refugiados ou requerentes de asilo.

### Características dos Estudos e suas Populações

Os 60 artigos selecionados foram todos publicados em inglês entre 1990 e 2017 (45% entre 2000 e 2010) e provenientes de 31 países diferentes (14 dos EUA). Os estudos eram de desenho transversal (Tabela 1), exceto dois estudos de coorte^48,73^.

**Tabela 1:** Características dos estudos incluídos na revisão sistemática e prevalências de violência sexual. (n = 60)

Primeiro autor e ano do estudo	País(es)/ região de acolhimento	Local de coleta de dados	Período de coleta de dados		Instrumento do estudo	Amostra (n)	Idade média da amostra (anos)	Proporção de sexo feminino (%)	Prevalência global de VS (%)*	Prevalência de VS por sexo	
	
			Início	Fim						Feminino	Masculino
Allodi^14^ (1990)	Canadá	USS	1979	1985	NI	56	NI	50,0	NI	64,3	39,3
Fornazzari^15^ (1990)	Canadá	USS	NI	NI	Coleta em registros	36	37	100,0	22,2	22,2	NA
Mckelvey^16^ (1995)	Filipinas	USS	NI	NI	QP	102	NI	33,3	9,8	8,8	10,3
Peel^17^ (1996)	Reino Unido	USS e centros de detenção	1993	1994	Coleta em registros	92	NI	21,7	33,7	80	20,8
Frljak^18^ (1997)	Bósnia e Herzegovina	USS	1993	1994	Coleta em registros	241	NI	100,0	3,3	3,3	NA
Silove^19^ (1998)	Austrália	NI	NI	NI	HTQ	96	NI	NI	0,0	0,0	0,0
Gorst-Unsworth^20^ (1998)	Reino Unido	USS	NI	NI	HTQ	84	39	0,0	14,3	NA	14,3
Loutan^21^ (1999)	Suíça	USS	1993	1994	HTQ	573	27	36,3	2,3	NI	NI
Blair^22^ (2000)	EUA	USS e domicílios	1991	1991	WTS	124	37	60,5	5,6	NI	NI
Hondius^23^ (2000)	Holanda	USS	NI	NI	NI	156	NI	34	23,1	26,4	21,4
Petersen^24^ (2000)	Tailândia	CR	1999	1999	QP	129	36	37,2	NI	6,3	NI
Iacopino^25^ (2001)	Macedônia e Albânia	CR	1999	1999	QP	11.458	NI	NI	0,03	NI	NI
Tang^26^ (2001)	Gâmbia	CR	1999	1999	HTQ	80	41,3	48,8	1,3	NI	NI
Crescenzi^27^ (2002)	Índia	Aldeias	1995	1995	HTQ	150	NI	37,3	NI	NI	NI
Sabin^28^ (2003)	México	CR	2000	2000	HTQ	170	37,9	58,2	3,5	NI	NI
Cardozo^29^ (2004)	Tailândia	CR	2001	2001	HTQ	495	NI	57,4	NI	2,8	2,9
Sesay^30^ (2004)	Serra Leoa	CR e aldeias	2001	2011	QP	400	NI	100,0	11,3	11,3	NA
Thomas^31^ (2004)	Reino Unido	NI	NI	NI	NI	100	16	41	32	63,4	10,2
Asgary^32^ (2006)	EUA	USS	1998	2002	Protocolo de Istambul	89	34	13,5	NI	NI	NI
Avdibegovic^33^ (2006)	Bósnia e Herzegovina	USS e CR	2000	2002	DVI modificado	50	NI	100,0	30,0	30,0	NA
Bradley^34^ (2006)	Reino Unido	USS	NI	NI	NI	97	30	14,4	8,2	28,6	2,4
Schweitzer^35^ (2006)	Austrália	Comunidade	2003	2003	HTQ	63	34,2	33,3	11,1	19	7,1
Olsen^36^ (2006)	Dinamarca	USS	1991	1994	QP	221	35,6	12,7	11,3	NI	NI
Bogner^37^ (2007)	Inglaterra	USS	2004	2005	QP	27	NI	59,3	55,6	68,8	36,4
Edston^38^ (2007)	Suécia	USS	1993	2005	NI	63	28	100,0	76,2	76,2	NA
Hammoury^39^ (2007)	Líbano	USS	2005	2005	AAS	349	28	100,0	26,4	26,4	NA
Hooberman^40^ (2007)	EUA	USS	2000	2003	HTQ	325	33,5	38,8	28,9	NI	NI
John-Langba^41^ (2007)	Botsuana	CR	NI	NI	SGBV	402	29,2	100,0	99,8	99,8	NA
Kira^42^ (2007)	EUA	NI	NI	NI	CTS	501	35,7	45,3	1,2	NI	NI
Piwowarczyk^43^ (2007)	EUA	USS	1999	2002	NI	134	34	65,7	50,0	NI	NI
Chang^44^ (2008)	EUA	USS	2001	2001	NI	243	10,6	51,9	4,9	NI	NI
Nagai^45^ (2008)	Uganda	CR e aldeias	1999	2000	QP	1.216	NI	78,0	NI	18,1	16,9
Harrison^46^ (2009)	Uganda	CR e aldeias	2006	2006	BSS	1.158	NI	52,4	NI	3,8	NI
Mitike^47^ (2009)	Etiópia	CR	2004	2004	QP	288	NI	100,0	42,4	42,4	NA
Williams^48^ (2010)	Reino Unido	USS	2005	2005	NI	178	30,4	35,4	25,8	54,0	10,4
Schubert^49^ (2011)	Finlândia	USS	NI	NI	HTQ	78	37,6	37,2	NI	NI	NI
Tamblym^50^ (2011)	EUA	USS	2004	2007	HTQ modificado	58	34,7	29,3	20,7	NI	NI
Bogic^51^ (2012)	Alemanha, Itália e Reino Unido	Domicílios e comunidades	2005	2006	LSC	854	41,6	51,3	5,2	NI	NI
Kira^52^ (2012)	EUA	NI	2006	2006	CTS	209	NI	0,0	90,9	NI	NI
Parmar^53^ (2012)	República dos Camarões	Aldeias	2010	2010	NI	191	35,1	100,0	40,8	40,8	NA
Black^54^ (2013)	EUA	USS e comunidade	2004	2004	CREV SECV	196	13,8	45,9	4,6	NI	NI
Falb^55^ (2013)	Tailândia	CR	2008	2008	RHA	861	30,1	100,0	NI	NI	NA
Tufan^56^ (2013)	Turquia	USS	2005	2007	SLESQ	67	30,6	41,8	20,9	46,4	2,6
Gibson-Helm^57^ (2014)	Austrália	USS	2002	2011	NI	1.279	NI	100,0	5,7	5,7	NA
Idemudia^58^ (2014)	Polokwane, África do Sul	Cidade	NI	NI	QP	125	28,3	42,3	NI	NI	NI
Morof^59^ (2014)	Uganda	NI	2010	2010	HTQ QP	117	31,6	100,0	71,8	71,8	NA
Bell^60^ (2015)	Ruanda	CR	2008	2008	RHA toolkit	810	29	100,0	8,0	8,0	NA
Connor^61^ (2015)	EUA	Comunidade	NI	NI	QP	30	31,8	100,0	93,3	93,3	NA
Sipsma^62^ (2015)	Ruanda	CR	NI	NI	RHA toolkit	548	32	100,0	38,1	38,1	NA
Al-Modallal^63^ (2016)	Jordânia	CR	NI	NI	AAS	238	32,7	100,0	21,0	21,0	NA
Chu^64^ (2016)	EUA	Comunidades e domicílios	2014	2014	QP	15	NI	100,0	60,0	60,0	NA
Lerner^65^ (2016)	EUA	USS	2010	2013	QP	267	34	33,0	33,3	NI	NI
Um^66^ (2016)	Coréia do Sul	NI	2010	2010	CTS2	180	39,8	100,0	25,6	25,6	NA
Wirtz^67^ (2016)	Etiópia	CR	2012	2012	ASIST-GBV	487	NI	100,0	NI	NI	NA
Gušić^68^ (2017)	Suécia	Escolas USS	NI	NI	WRGTI	77	NI	35,0	12,0	NI	NI
Hopkinson^69^ (2017)	EUA	USS	2008	2013	HTQ QP	61	28,8	37,7	62,3	NI	NI
Logie^70^ (2017)	Canadá	Comunidades e redes sociais	2013	2015	QP	42	NI	100,0	52,0	52,0	NA
Riley^71^ (2017)	Bangladesh	CR	NI	NI	HTQ	148	34	52,8	13,0	NI	NI
Stark^72^ (2017)	Etiópia	CR	2015	2015	NI	919	14,6	100,0	65,3	65,3	NA
Wright^73^ (2017)	EUA	Agências dereassentamento	2011	2012	HTQ	298	NI	45,0	NI	1,5	NI

VS: violência sexual; NA: não se aplica; NI: não informado; CR: campos de refugiados; EUA: Estados Unidos da América; USS: unidades de serviços de saúde; QP: questionário da pesquisa; HTQ: *Harvard Trauma Questionnaire*; STAR: *Resettlement Stressor Scale*; WTS: *War Trauma Scale*; DVI: *Domestic Violence Inventory*; AAS: *Abuse Assessment Screen*; SBGV: *Sexual and Gender-based Violence Scale*; CTS: *Revised Conflict Tactics Scales*; CREV: *Children’s Report of Exposure to Violence*; SECV: *Survey of Exposure to Community Violence*; BSS: *Behavioral Surveillance Surveys Questionnaire*; SLESQ: *Stressful Life Events Screening Questionnaire*; LEC: *Life Events Checklist*; ASIST-GBV: *Assessment Screen to Identify Survivors Toolkit for Gender Based Violence*; LSC: *Life Stressor Checklist*; RHA: *Reproductive Health Assessment*; WRGTI: *War/refugee and general trauma inventory*

* A prevalência de VS global foi calculada a partir do total de casos informados pelos estudos ou, quando não havia essa informação, pela soma total dos casos específicos relatados (ex.: casos de estupro, casos de assédio sexual, etc.). Porém, em cinco estudos^32,49,55,58,67^, a prevalência global não pôde ser estimada, pois os autores não informaram o número total de casos. Não foi possível calculá-la a partir da soma das prevalências tipificadas, porque houve vítimas que sofreram mais de um tipo de VS, o que superestimaria o cálculo da prevalência global.

Os locais mais frequentes da coleta de dados, conforme os 54 artigos que continham essa informação, foram os serviços de saúde (n = 28,52%) e campos de refugiados (n = 17,32%). A maior parte dos estudos (87%) foi conduzida para avaliar desfechos em saúde mental, sem o objetivo principal de mensurar a prevalência de casos de VS. Dentre 49 que informaram o instrumento utilizado, o *Harvard Trauma Questionnaire* (HTQ) foi o instrumento validado mais frequente (n = 15, correspondendo a 31%), enquanto 29% (n = 14) utilizaram questionários elaborados especificamente para a pesquisa.

Os estudos envolveram 28.101 refugiados e requerentes de asilo. A população de cada estudo variou entre 15 e 11.458 indivíduos. Em 33% (n = 20) dos estudos, a amostra incluiu menos de 100 pessoas, e em 18% (n = 11), mais de 500 pessoas. A média da idade dos participantes variou entre 10,6 e 41,6 anos; 42% (n = 25) dos estudos incluíram menores de 18 anos. Houve predominância geral de mulheres; em 37% (n = 21) dos estudos, a amostra era exclusivamente de mulheres. A religião predominante foi a muçulmana, em 12 (63%) dos 19 estudos com dados a respeito.

### Prevalência de Violência Sexual

A variação da prevalência global apresentou grande amplitude, independentemente do tamanho da amostra: de 0% até 99,8%, com um total de 2.859 casos de VS. Em 15 estudos (31%), a prevalência foi menor que 10% (amostras de 80 a 11.458 pessoas), e em 11 (23%), maior que 50% (amostras de 15 a 919 pessoas), conforme demonstrado na Tabela 1. Essa ampla variação ocorreu independentemente do cenário da coleta de dados – em campos de refugiados (n = 12; 0,03% a 99,8%), unidades de saúde (n = 25; 2,3% a 76,2%) e comunidades/aldeias (n = 6; 5,2% a 93,3%) – e da forma de mensuração – instrumentos validados (n = 25; 0,0% a 99,8%) ou questionários próprios da pesquisa (n = 14; 0,03% a 93,3%).

Seis estudos relataram VS em crianças e adolescentes, com prevalências variando entre 4,6% e 90,9%^16,44,47,52,54,72^. Em 32 dos 36 (89%) estudos que apresentaram prevalências por sexo, as principais vítimas foram mulheres. Desses, 12 estudos relataram VS nos dois sexos, com uma diferença de até 59,2% a mais na prevalência em mulheres^17^. Dois estudos relataram o oposto, mas com disparidades menores que 2%^16,29^. Em homens, a prevalência atingiu 39,3%^14^.

A África foi o continente de origem mais frequente em 13 (42%) dos 31 estudos com informações a respeito (Tabela 2). Quanto ao momento da ocorrência, abordado por 18 estudos, 17 (94%) relataram que a VS ocorreu no país de origem (prevalências entre 1% e 92%); em dois estudos (11%), aconteceu durante o percurso (prevalências de 5,2% em ambos)^53,68^; e dois (11%) relataram VS no local de acolhimento (prevalências de 39% na República dos Camarões^53^ e 46,1% em Uganda^59^).

**Tabela 2:** Prevalência de violência sexual em refugiados segundo o lugar de origem. (n = 31)

Continente(s) de origem	Região de origem	País(es) de origem	Primeiro autor e ano do estudo	Amostra (n)	Prevalência de VS
África (n = 13)	NI	NI	Thomas^31^ (2004)	65	24,6
	NI	NI	Chu^64^ (2016)	15	60,0
	África Central	RDC	Peel^17^ (1996)	92	33,7
		RDC	Edston^38^ (2007)	3	100,0
		República Centro-Africana	Parmar^53^ (2012)	77	57,1
		RDC	Bell^60^ (2015)	810	8,0
		RDC	Sipsma^62^ (2015)	548	38,1
	África Ocidental	Senegal	Tang^26^ (2001)	80	1,3
		Serra Leoa	Sesay^30^ (2004)	400	11,3
		NI	Gibson-Helm^57^ (2014)	45	6,7
	Norte da África	Sudão	Schweitzer^35^ (2006)	63	11,1
		Sudão e Sudão do Sul	Stark^72^ (2017)	919	65,3
		NI	Gibson-Helm^57^ (2014)	1.147	5,1
	África Oriental	Uganda	Edston^38^ (2007)	9	66,7
		Somália	Mitike^47^ (2009)	248	49,2
		NI	Gibson-Helm^57^ (2014)	87	13,8
Ásia (n = 8)	Ásia Meridional	Sri Lanka	Silove^19^ (1998)	92	0,0
		Bangladesh	Edston^28^ (2007)	13	84,6
	Sul da Ásia	Mianmar	Petersen^24^ (2000)	129	2,3
		Mianmar	Riley^71^ (2017)	148	13,0
	Sudeste Asiático	Vietnã	McKelvey^16^ (1995)	102	9,8
		Camboja	Blair^22^ (2000)	124	5,6
		Camboja	Chang^44^ (2008)	243	4,9
	Ásia Oriental	Coréia do Norte	Um^66^ (2016)	180	25,6
Europa Ásia África (n = 8)	Oriente Médio	NI	Olsen^36^ (2006)	221	11,3
		NI	Wright^73^ (2017)	133	1,5
Europa Ásia África (n = 8)	Oriente Médio	Iraque	Gorst-Unsworth^20^ (1998)	84	14,3
		Iraque	Kira^42^ (2007)	501	1,2
		Iraque	Kira^52^ (2012)	209	90,9
		Iraque	Black^54^ (2013)	196	4,6
		Irã	Edston^38^ (2007)	11	45,5
		Síria	Edston^38^ (2007)	3	66,7
		Turquia	Bradley^34^ (2006)	97	8,2
		Turquia	Edston^38^ (2007)	3	100,0
NA (n = 2)	Palestina	NA	Hammoury^39^ (2007)	349	26,4
		NA	Al-Modallal^63^ (2016)	238	21,0
América (n = 1)	América Central	Guatemala	Sabin^28^ (2003)	170	3,5
Europa (n = 1)		Bósnia	Frljak^18^ (1997)	241	3,3

VS: violência sexual; NI: não informado; NA: não se aplica; RDC: República Democrática do Congo

O tipo de VS mais frequente foi o estupro (65%) (Tabela 3). Os perpetradores foram identificados em 18 estudos: 10 (55%) relataram a ocorrência de VS por parceiro íntimo (prevalências de 4,3% a 30%)^33,39,45,53,55,59,62,63,66,72^, cinco por militares (prevalências de 1% a 74,6%)^38,45,55,58,72^, quatro por conhecidos^51,53,55,72^, quatro por familiares^45,54,58,72^, dois por desconhecidos^51,53^, dois por soldados rebeldes^31,53^, um por policiais^58^, um por grupos armados^72^ e um por guardas na prisão^17^.

**Tabela 3:** Prevalência de acordo com o tipo de violência sexual em refugiados. (n = 51)

Tipo de violência sexual	Primeiro autor e ano do estudo	Continente/região/país de origem	País/região de acolhimento	Prevalência (%)
Estupro (n = 33)	Allodi^14^ (1990)	América Latina	Canadá	30,4
	Fornazzari^15^ (1990)	América Latina	Canadá	22,2
	Peel^17^ (1996)	RDC	Reino Unido	33,7
	Frljak^18^ (1997)	Bósnia	Bósnia	3,3
	Silove^19^ (1998)	Sri Lanka	Austrália	0,0
	Loutan^21^ (1999)	África, Ásia e Europa	Suíça	2,3
	Petersen^24^ (2000)	Mianmar	Tailândia	2,3
	Tang^26^ (2001)	Senegal	Gâmbia	1,3
	Crescenzi^27^ (2002)	Tibete	Índia	0,7
	Cardozo^29^ (2004)	Mianmar	Tailândia	2,8
	Sesay^30^ (2004)	Serra Leoa	Serra Leoa	11,3
	Thomas^31^ (2004)	África, Oriente Médio, Europa Ocidental e Ásia	Reino Unido	32,0
	Asgary^32^ (2006)	África e Ásia	EUA	6,7
	Bradley^34^ (2006)	Turquia	Reino Unido	1,0
	Schweitzer^35^ (2006)	Sudão	Austrália	11,1
	Avdibegovic^33^ (2006)	NI	Bósnia	34,0
	Bogner^37^ (2007)	Oriente Médio, Europa, África e América Latina	Inglaterra	44,4
	Edston^38^ (2007)	África, Ásia e Oriente Médio	Suécia	76,2
	Hammoury^39^ (2007)	Palestina	Líbano	26,4
	Hooberman^40^ (2007)	África, Ásia, Europa e América Central e do Sul	EUA	18,2
	Harrison^46^ (2009)	África	Uganda	2,0
	Williams^48^ (2010)	África e Oriente Médio	Reino Unido	16,3
Estupro (n = 33)	Schubert^49^ (2011)	Oriente Médio, Sudeste da Europa, Sul da Ásia e África Central	Finlândia	21,8
	Kira^52^ (2012)	Iraque	EUA	90,9
	Falb^55^ (2013)	Mianmar	Tailândia	0,3
	Morof^59^ (2014)	Somália e RDC	Uganda	54,7
	Idemudia^58^ (2014)	Zimbábue	Polokwane, África do Sul	56,8
	Bell^60^ (2015)	RDC	Ruanda	8,0
	Lerner^65^ (2016)	África, América e Europa Ocidental	EUA	33,3
	Wirtz^67^ (2016)	Somália	Etiópia	20,1
	Hopkinson^69^ (2017)	África, Ásia, América e Europa Oriental	EUA	42,6
	Logie^70^ (2017)	NI	Canadá	52,0
	Stark^72^ (2017)	Sudão e Sudão do Sul	Etiópia	16,1
Toque sexual indesejado (n = 7)	Asgary^32^ (2006)	África e Ásia	EUA	6,7
	Avdibegovic^33^ (2006)	NI	Bósnia	2,0
	Schubert^48^ (2011)	Oriente Médio, Sudeste da Europa, Sul da Ásia e África Central	Finlândia	46,2
	Falb^55^ (2013)	Sudeste Asiático	Tailândia	0,7
	Idemudia^58^ (2014)	Zimbábue	Polokwane, África do Sul	63,2
	Hopkinson^69^ (2017)	África, Ásia, América e Europa Oriental	EUA	24,6
	Stark^72^ (2017)	Sudão e Sudão do Sul	Etiópia	22,0
Coerção sexual (n = 1)	Stark^72^ (2017)	Sudão e Sudão do Sul	Etiópia	27,3
Tentativa de estupro (n = 2)	Idemudia^58^ (2014)	Zimbábue	Polokwane, África do Sul	44,8
	Morof^59^ (2014)	Somália e RDC	Uganda	64,1
Gravidez forçada (n = 1)	Wirtz^67^ (2016)	África Oriental	Etiópia	15,6
Tortura sexual (n = 6)	Hondius ^23^ (2000)	Turquia e Irã	Holanda	23,1
	Asgary^32^ (2006)	África e Ásia	EUA	9,0
	Bradley^34^ (2006)	Turquia	Reino Unido	2,1
	Olsen^36^ (2006)	Oriente Médio	Dinamarca	11,3
	Bogner^37^ (2007)	Oriente Médio, Europa, África e América Latina	Inglaterra	11,1
	Tamblyn^50^ (2011)	África	EUA	20,7
Agressão sexual (n = 5)	Gorst-Unsworth^20^ (1998)	Iraque	Reino Unido	14,3
	Iacopino^25^ (2001)	Kosovo	Macedônia	0,03
	Bradley^34^ (2006)	Turquia	Reino Unido	8,2
	Hooberman^40^ (2007)	África, Ásia, América Central e do Sul e Europa	EUA	10,8
	Williams^48^ (2010)	África e Oriente Médio	Reino Unido	12,9
Mutilação genital (n = 6)	Asgary^32^ (2006)	África e Ásia	EUA	2,2
	Bradley^34^ (2006)	Turquia	Reino Unido	1,0
	Mitike^47^ (2009)	Somália	Etiópia	42,4
	Gibson-Helm^57^ (2014)	África e Oriente Médio	Austrália	5,7
	Connor^61^ (2015)	Somália e Etiópia	EUA	93,3
	Chu^64^ (2016)	África	EUA	60,0
Exploração sexual (n = 4)	Cardozo^29^ (2004)	Mianmar	Tailândia	1,0
	Nagai^45^ (2008)	Sudão	Uganda	82,0
	Idemudia^58^ (2014)	Zimbábue	África do Sul	44,0
	Wirtz^67^ (2016)	Somália	Etiópia	27,3
Experiência sexual indesejada sem contato (n = 5)	Crescenzi^27^ (2002)	Tibete	Índia	24,6
	Asgary^32^ (2006)	África e Ásia	EUA	4,5
	Avdibegovic^33^ (2006)	NI	Bósnia	2,0
	Falb^55^ (2013)	Mianmar	Tailândia	1,5
	Hopkinson^69^ (2017)	África, Ásia, América, Europa Oriental	EUA	29,8
Abuso sexual (n = 8)	Allodi^14^ (1990)	América Latina	Canadá	21,4
	McKelvey^16^ (1995)	Vietnã	Filipinas	9,8
	Blair^22^ (2000)	Camboja	EUA	5,6
	Kira^42^ (2007)	Iraque	EUA	1,2
	Chang^44^ (2008)	Camboja	EUA	4,9
	Nagai^45^ (2008)	Sudão	Uganda	85,0
	Black^54^ (2013)	Iraque	EUA	4,6
	Riley^71^ (2017)	Mianmar	Bangladesh	13,0
Casamento forçado (n = 2)	Asgary^32^ (2006)	África e Ásia	EUA	2,2
	Wirtz^67^ (2016)	Somália	Etiópia	19,5
Assédio sexual (n = 4)	Asgary^32^ (2006)	África e Ásia	EUA	12,4
	Bogic^51^ (2012)	Bósnia	Alemanha, Itália, Reino Unido	5,2
	Idemudia^58^ (2014)	Zimbábue	Polokwane, África do Sul	52,8
	Wright^73^ (2017)	Oriente Médio	EUA	1,5

NI: não informado; EUA: Estados Unidos da América; RDC: República Democrática do Congo

Em cinco estudos^32,49,55,58,67^, os autores não informaram o número de vítimas, e não foi possível estimar a prevalência global. A estimativa pela soma das prevalências por tipo específico superestimaria a prevalência global em razão dos casos que sofreram mais de um tipo de VS.

## DISCUSSÃO

Estudos prévios haviam revelado que a VS é uma ameaça constante ao longo de todo o percurso migratório dos refugiados^3,12,13^, o que foi confirmado na presente revisão. Apesar de a maioria dos estudos aqui identificados terem revelado uma maior prevalência entre mulheres adultas, a VS também constituiu um sério problema em homens e crianças. Ademais, foi observado que a VS é perpetrada principalmente por parceiros íntimos, mas também por militares, guardas e policiais. A maior parte dos casos ocorre no país de origem, na forma de estupro e em refugiados provenientes da África. Em alguns campos de refugiados, como em Uganda e República dos Camarões, a frequência foi alarmante.

É possível que as prevalências estejam subestimadas em alguns estudos, pois muitas vítimas – principalmente homens – não relatam a VS por vergonha, ameaças por parte dos perpetradores, medo de serem consideradas culpadas ou de sofrer estigma e exclusão da família e comunidade^6,74^, com consequente baixa procura por atendimento de saúde e registro dos casos^75^. Além disso, a crise humanitária ocasionada pelos conflitos armados nos países de origem dos refugiados gera grandes deslocamentos de pessoas e demandas incompatíveis com a disponibilidade de serviços e recursos de saúde^76^, o que pode reduzir ainda mais as chances de identificação de casos. Por outro lado, os estudos focados na avaliação de trauma mental em serviços de saúde possivelmente superestimam a prevalência.

Na meta-análise de prevalência de VS em mulheres nos cenários de complexos humanitários de emergência, que também incluiu deslocados internos e excluiu mutilação genital, a prevalência sumarizada foi de 21,4% e maior em refugiados provenientes da África^12^. Em nossa revisão, encontramos vários estudos com prevalências bastante superiores. Independentemente da real prevalência, a VS foi frequente nas populações estudadas, e merece atenção especial nos serviços de saúde e de acolhimento dessa população já fragilizada por traumas de guerra e perseguição.

As mulheres jovens são as principais vítimas de VS, mas homens, crianças e adolescentes também são vítimas, uma realidade pouco discutida na literatura. Homens e menores desacompanhados também estão expostos ao risco de exploração e abuso sexual durante a migração e na chegada aos países de destino^3^. Não obstante, a predominância em mulheres não causa surpresa. O processo de imigração é acompanhado por dificuldades como insegurança econômica, barreiras de linguagem e aculturação que levam ao desequilíbrio de poder entre mulheres e parceiros, provocando aumento das tensões^77^. Em virtude das mudanças econômicas, políticas e sociais durante guerras e pós-guerras, muitos homens utilizam violência para controlar as mulheres e reestabelecer seu status de poder^78^. Tais condições podem explicar a maior frequência de VS perpetrada por parceiros íntimos.

A VS ocorre principalmente antes da migração, nos países de origem dos refugiados. Isso sugere uma relação com as condições geradas pelos conflitos armados, que potencializam normas culturais de superioridade do poder masculino presentes nesses locais, antes mesmo da condição de busca de refúgio. As elevadas prevalências na África corroboram essa visão. A República Democrática do Congo, onde conflitos armados em torno das reservas de recursos naturais perduram desde a independência em 1960^79^, é marcada por atrocidades que incluem estupro por grupos, escravidão sexual, participação forçada de familiares em estupros, mutilação genital, entre outras^80^. Mais chocante é o fato de que, mesmo quando acolhida em campos de refugiados, essa população, já fragilizada, ainda enfrenta insegurança e sofre VS perpetrada por aqueles de quem esperam proteção, como oficiais e policiais.

O estupro foi a forma mais mencionada dessa violência. Isso pode ser explicado pela definição mais concreta, pela experiência mais marcante e porque grande parte dos estudos utilizou o instrumento HTQ, que possui uma questão específica sobre estupro e abuso sexual, mas não sobre outras formas de VS. O estupro é considerado o tipo mais cruel por trazer graves e sérias consequências à saúde das vítimas. Sobreviventes de guerra com diagnóstico de transtorno de estresse pós-traumático e vítimas de estupro relatam mais sintomas somáticos que aqueles sem experiência de estupro^81^. O estupro também aumenta as chances de adquirir infecção por HIV, como relatado em mulheres refugiadas da África subsaariana em Paris, e está relacionado com dificuldades sociais e falta de residência fixa devido ao risco de sofrer sexo transacional ou assédio sexual durante a hospedagem por parentes ou conhecidos^82^.

Vários estudos incluídos nesta revisão apresentaram diversas limitações, como a falta de detalhamento sobre a população, desfecho de interesse, momento da ocorrência, perfil dos perpetradores, sexo e idade das vítimas. Além disso, os estudos não incluíram vítimas da crise migratória mais recente, iniciada em 2015.

Nossa revisão também apresenta limitações. A busca bibliográfica não incluiu os termos “tortura sexual” e “mutilação genital”, o que pode ter resultado em baixa sensibilidade e explicar a quantidade de artigos encontrados nas listas de referências. Não incluímos a literatura cinzenta e não foi realizada avaliação da qualidade metodológica dos estudos selecionados. Ademais, não restringimos o tamanho da amostra dos artigos, o que resultou em estimativas imprecisas em estudos com poucos indivíduos^38^. Finalmente, as diferenças metodológicas entre os estudos (diferentes locais de coleta de dados, como serviços de saúde mental e campos de refugiados; diferentes instrumentos de coleta de dados; estudos focados em transtornos mentais e não na prevalência de VS; e amostragens desiguais) podem ter contribuído para a diversidade das taxas encontradas e heterogeneidade entre os estudos, o que impediu a realização de uma meta-análise para sumarizar as informações.

Em resumo, os resultados desta revisão mostram que a VS é um problema frequente entre refugiados, tanto em mulheres quanto em homens, principalmente vindos da África, que ocorre em todos os momentos do processo migratório, inclusive nos locais de suposto acolhimento. O problema da VS entre os refugiados da crise migratória mais recente precisa ser investigado, em cenários não selecionados e com métodos mais adequados para melhor orientar as medidas de proteção necessárias.
